# Binaural Beat: A Failure to Enhance EEG Power and Emotional Arousal

**DOI:** 10.3389/fnhum.2017.00557

**Published:** 2017-11-15

**Authors:** Fran López-Caballero, Carles Escera

**Affiliations:** ^1^Brainlab-Cognitive Neuroscience Research Group, Department of Clinical Psychology and Psychobiology, University of Barcelona, Barcelona, Spain; ^2^Institute of Neurosciences, University of Barcelona, Barcelona, Spain; ^3^Institut de Recerca Sant Joan de Déu (IRSJD), Barcelona, Spain

**Keywords:** binaural beats, acoustic beats, EEG bands, heart rate, skin conductance

## Abstract

When two pure tones of slightly different frequencies are delivered simultaneously to the two ears, is generated a beat whose frequency corresponds to the frequency difference between them. That beat is known as acoustic beat. If these two tones are presented one to each ear, they still produce the sensation of the same beat, although no physical combination of the tones occurs outside the auditory system. This phenomenon is called binaural beat. In the present study, we explored the potential contribution of binaural beats to the enhancement of specific electroencephalographic (EEG) bands, as previous studies suggest the potential usefulness of binaural beats as a brainwave entrainment tool. Additionally, we analyzed the effects of binaural-beat stimulation on two psychophysiological measures related to emotional arousal: heart rate and skin conductance. Beats of five different frequencies (4.53 Hz -theta-, 8.97 Hz -alpha-, 17.93 Hz -beta-, 34.49 Hz -gamma- or 57.3 Hz -upper-gamma) were presented binaurally and acoustically for epochs of 3 min (Beat epochs), preceded and followed by pink noise epochs of 90 s (Baseline and Post epochs, respectively). In each of these epochs, we analyzed the EEG spectral power, as well as calculated the heart rate and skin conductance response (SCR). For all the beat frequencies used for stimulation, no significant changes between Baseline and Beat epochs were observed within the corresponding EEG bands, neither with binaural or with acoustic beats. Additional analysis of spectral EEG topographies yielded negative results for the effect of binaural beats in the scalp distribution of EEG spectral power. In the psychophysiological measures, no changes in heart rate and skin conductance were observed for any of the beat frequencies presented. Our results do not support binaural-beat stimulation as a potential tool for the enhancement of EEG oscillatory activity, nor to induce changes in emotional arousal.

## Introduction

When two pure-tone sinewaves with slightly different frequencies (e.g., 300 and 305 Hz) are presented simultaneously to the same ear, a periodic two-tone complex with a frequency corresponding to the frequency difference between the two tones (e.g., 5 Hz) can be perceived as a “beat”. In such a phenomenon, known as “acoustic beat”, the two input frequencies are physically mixed in the signal before they reach the auditory system. In contrast, when the same two tones with slightly different frequencies are played binaurally, one to each ear, the same beat is perceived, although no physical combination of these tones occurs outside the auditory system, hence pointing to a central nervous origin. This latter effect is known as the “binaural beat”, and can be perceived if the carrier frequency is lower than 1000 Hz, and the two frequencies differ from each other by approximately less than 35 Hz (Licklider et al., 1950).

First described by Dove (1841) and further characterized by Thompson (1877), binaural-beat phenomena reflect the convergence of neural activity from the auditory nerves in binaurally sensitive networks (Moore, 1997). There is agreement on the involvement of the auditory cortex and the brainstem in the neural mechanisms behind binaural beats perception. In animal studies, single-unit recordings have disclosed the earliest responses evoked by binaural-beat stimulation in neurons of the superior olivary complex of the brainstem (Wernick and Starr, 1968; Spitzer and Semple, 1998), the first nucleus in the ascending auditory pathway receiving bilateral input. Moreover, these studies have disclosed responses in the inferior colliculus of the midbrain that are phase-locked to the binaural-beat frequency (Kuwada et al., 1979; McAlpine et al., 1996).

In humans, magnetoencephalographic (MEG) studies have recorded auditory steady-state responses to binaural beats from various sources in the parietal, frontal and temporal areas of the cerebral cortex, including the auditory cortices (Karino et al., 2006). Moreover, similar studies have suggested the involvement of the medial superior olive and the inferior colliculus in their generation mechanism (Draganova et al., 2008). In contrast with binaural beats, neuronal correlates of acoustic beats, physically present in the acoustic signal, have been found in the cochlear nuclei, the earliest relay of the auditory pathway (Draganova et al., 2008). Interestingly, at cortical level, results from electroencephalographic (EEG) studies with event-related potentials have suggested a similar involvement of the cortex in the processing of both acoustic and binaural beats (Pratt et al., 2010). Altogether, these studies suggest that binaural beat perception is the result of the integration of auditory signals from each ear in the superior olivary complex and the inferior colliculus, with a resulting neuroelectrical discharge that travels along the brainstem up to the auditory cortex.

A controversial aspect of binaural-beat research is their claimed benefit for the enhancement of specific brain wave oscillatory activity. Such research field acquires particular relevance as the traditional EEG frequency bands, such as theta (4–7 Hz), alpha (8–12 Hz), beta (13–30 Hz) and gamma (30–100 + Hz) have been associated with specific cognitive functions, such as selective attention and memory (for a review, see Herrmann et al., 2016), and the modulation or entrainment of neural oscillatory activity may constitute an effective approach for their enhancement (Huang and Charyton, 2008). As the human hearing range excludes frequencies below 20 Hz, which fall within the range of some of the relevant EEG bands for cognitive enhancement, stimulation with binaural beats has been suggested as a potential beneficial tool to alter brainwave rhythmicity in a non-invasive manner (for a review, see Vernon, 2009).

Some empirical studies have addressed the intriguing question whether binaural beats could indeed modulate specific brain rhythms leading to enhanced cognitive functions, yet with contradictory results. From studies examining the effects of binaural beats in cognitive functions, binaural-beat stimulation with frequencies within EEG theta band has resulted in no effects on vigilance task performance (Goodin et al., 2012), whereas binaural-beat stimulation with “beta” frequencies has been shown to increase performance in tasks related to verbal span, working memory, executive functions (Kennerly, 1996) and vigilance (Lane et al., 1998). Moreover, stimulation with binaural beats with frequencies in the range of the EEG gamma band affects divergent thinking (Reedijk et al., 2013) and attentional control (Reedijk et al., 2015).

Studies addressing the effects of binaural-beat stimulation on neuroelectric brain activity have reported induced oscillatory activity in the EEG theta band after binaural-beat stimulation within this very same range (Brady and Stevens, 2000), although subsequent experiments failed to replicate these effects (Stevens et al., 2003; Wahbeh et al., 2007; Gao et al., 2014). Also, other studies failed to elicit entrainment of EEG oscillatory activity to binaural alpha-beats (Gao et al., 2014; Vernon et al., 2014), as well as to binaural beta-beats (Goodin et al., 2012; Gao et al., 2014; Vernon et al., 2014). Furthermore, when using beats with frequencies within EEG gamma band, some studies revealed induced EEG gamma-band oscillatory activity with both binaural (Lavallee et al., 2011; Ross et al., 2014) and acoustic (Ross et al., 2014) beat stimulation. Entrainment effects were also reported in EEG gamma band with acoustic beats within gamma frequencies in intracranial recordings (Becher et al., 2015). In addition to induced activity at EEG bands, several studies have addressed binaural-beat effects on EEG evoked activity. Ioannou et al. (2015) recorded auditory steady-state responses to binaural alpha-beats but not to binaural gamma-beats, whereas the already cited MEG study by Draganova et al. (2008) reported auditory steady-state responses to binaural gamma-beats.

Beyond EEG entrainment and cognitive enhancement, binaural-beat stimulation has also been related with other clinically relevant dimensions, such as parasympathetic activation and self-reported relaxation (McConnell et al., 2014), heart rate variability (Palaniappan et al., 2015) and acute pre-operative anxiety (Padmanabhan et al., 2005), for theta-, alpha- and delta-beat stimulation, respectively. On the other hand, a recent comprehensive review concludes a diminishing impact of binaural-beat stimulation on anxiety levels (Chaieb et al., 2015). Overall, stimulation with binaural beats could be a tool not only to entrain brain rhythms, but also to induce changes in autonomic functions, which may be helpful in clinical populations such as those suffering from hypertension, sleep or anxiety disorders, among others.

From the studies reviewed above, regarding the effects of binaural beats on electrophysiological, cognitive and affective measures, no conclusions can be drawn. The different experimental protocols in these studies, including stimulus duration, the specific beat frequencies used within the same range, the participant’s attention to the stimuli (Schwarz and Taylor, 2005), as well as individual differences (Reedijk et al., 2015) may account for the observed contradictory results. In addition, in several of the studies, the lack of proper control conditions, the lack of details about the experimental protocols, as well as the fact that binaural-beat effects on cognitive processes were assessed without monitoring brain oscillatory activity, result in methodological limitations that leave the issue controversial. In fact, electrophysiological research comparing the effects of beat stimulation under different conditions is still rare (Chaieb et al., 2015). On the other hand, from the reviewed literature it is also under debate the role of binaural beat stimulation in modulating autonomic functions. Disentangling such role may help to prove the potential clinical effectiveness of binaural beats.

The goal of the present study was therefore to elucidate whether binaural-beat stimulation at different beat frequencies would affect EEG oscillatory activity at the particular frequency of the beat stimulation, to test the potential usefulness of binaural beats as a brainwave entrainment tool. Particularly, we addressed this question by using a paradigm that allowed us to disentangle the specific and differential contribution of binaural beats over that of acoustic beats. Despite the exploratory nature of the present study and the inconclusive evidence on the topic, our theoretical prediction was that binaural beat stimulation would induce an enhancement of EEG power at the specific frequency of the beat. A secondary goal was to explore the effects of binaural-beat at different frequencies on psychophysiological measurements traditionally related to emotional arousal, such as heart rate and skin conductance, to test their suggested potential effect on autonomic function (McConnell et al., 2014) and thus further understand their potential clinical usefulness.

## Materials and Methods

### Participants

Fourteen participants (five males), ranging in age from 20 to 31 years (mean = 23.3; standard deviation = 3.3) were recruited among University of Barcelona students, and compensated by monetary payment (8 €/h). Exclusion criteria for the selection of participants were history of neurologic or psychiatric condition, as well as abnormal hearing. A pure-tone audiometry (frequency range: 250–4000 Hz), using audiometric Beyerdynamic DT48-A headphones (Heilbronn, Germany), was performed for each participant before the experiment started, ensuring mean hearing thresholds below 20 dB NHL at each ear. The experimental protocol was approved by the Bioethics Committee of the University of Barcelona, and was in accordance with the WMA Declaration of Helsinki Ethical Principles for Medical Research Involving Human Subjects. Before the experimental sessions, written informed consent was obtained from each participant after all the details of the research (except the hypotheses) were explained to them, including the characteristics of the EEG method and the possibility to withdraw from the experiment at their wish.

### Stimuli

**Figure 1:**
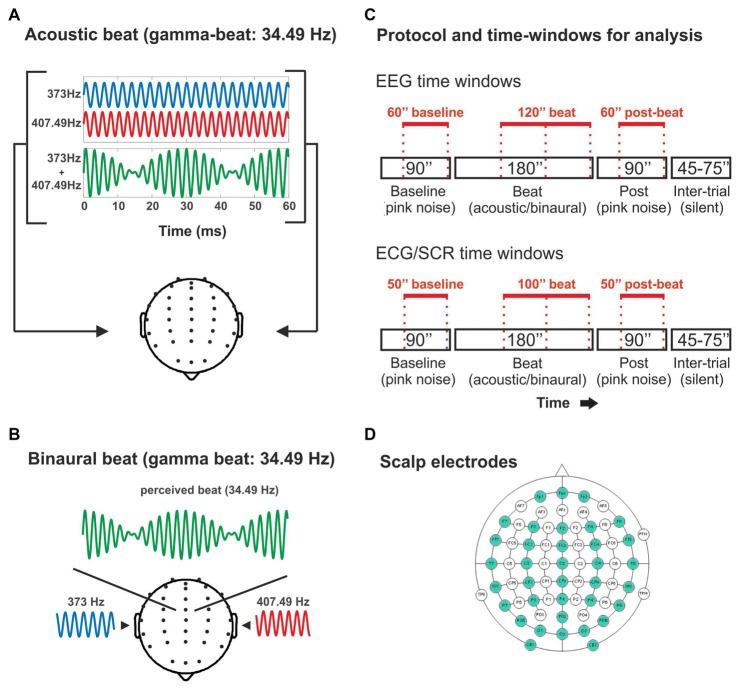
**(A)** Illustration of the acoustic-beat condition with a beat frequency within the electroencephalographic (EEG) gamma range. Two pure tones of slightly different frequencies (373 Hz and 407.49 Hz) were presented simultaneously to the two ears. Consequently, a beat is generated whose frequency corresponds to the frequency difference between them (34.49 Hz). **(B)** Illustration of the binaural-beat condition with a beat frequency within the EEG gamma range. One pure tone (373 Hz) is presented to one ear while another (407.49 Hz) is presented to the other ear. Thus, a beat whose frequency corresponds to the frequency difference between them (34.49 Hz) is generated within the auditory pathway. **(C)** Experimental protocol and time-windows for analysis. Experimental blocks consisted on 90 s of pink noise, followed by 180 s of acoustic or binaural beat at the corresponding frequency, followed in turn by 90 s of pink noise. In red, the time windows used for the analyses of the EEG band power (EEG: above) and psychophysiological measures (electrocardiogram, ECG and skin conductance response, SCR: below). **(D)** Scalp electrodes used in the EEG recording (in green).

Stimuli were pure tones delivered at 75 dB SPL. Acoustic and binaural beats were generated by presenting two pure tones with slightly different frequencies, either simultaneously to both ears (acoustic beat) or separately with one tone to each ear (binaural beat; Figures 1A,B). The frequency of one of the pure tones in all conditions was set to 373 Hz, and the beats were created by adding a second sinewave differing from the first in 4.53 Hz (theta-beat), 8.97 Hz (alpha-beat), 17.93 Hz (beta-beat), 34.49 Hz (gamma-beat) or 57.3 Hz (upper gamma-beat; exceeding the frequency limit above which the perception of binaural beats was suggested to be not possible; Licklider et al., 1950). We selected these frequency values as an intermediate point within the typical EEG frequency bands. Particularly, we set for the gamma-beat to be below 35 Hz because of the already mentioned limitation to the perception of binaural beats (Licklider et al., 1950). Besides, we included an “upper gamma”-beat to surpass this limit, in order to test the effects on the EEG of a beat that cannot be perceived. All stimuli were created and presented using MATLAB software (The Mathworks, Inc., Natick, MA, USA) and the Psychophysics Toolbox extensions (Brainard, 1997; Pelli, 1997; Kleiner et al., 2007).

### Procedure and Experimental Design

The EEG experiments were conducted on a single 3-h session, including subject preparation and recording. During the experimental sessions, participants were seated comfortably in an electrically shielded and sound-attenuated chamber, while passively listening to the auditory stimuli presented through ER-3A ABR insert earphones (Etymotic Research, Inc., Elk Grove Village, IL, USA). Participants were instructed to watch a silent movie with subtitles.

There were two conditions: *binaural-beat*, in which binaural beats in one of the five frequencies were presented, and *acoustic-beat*, in which acoustic beats of the same five frequencies were presented instead. Stimuli were presented in 10 blocks lasting 6 min each (Figure 1C): one acoustic and one binaural block for each of the five beat frequencies of the study. Each block started with 90 s of a constant background pink noise (65 dB SPL), followed by 180 s of acoustic- or binaural-beat stimulation delivered +10 dB over the pink noise (i.e., 75 dB SPL), and subsequently followed by 90 additional seconds of pink noise (65 dB SPL). Binaural or acoustic beats were presented in a continuous fashion. Between blocks, there were from 45 s to 75 s of silence. The order of the blocks was randomized across participants, with the only restriction that two blocks with the same frequency could not be presented in a row (e.g., binaural theta-beat preceded by acoustic theta-beat).

### Data Acquisition

Continuous EEG recordings were carried out from 36 scalp electrodes (Fp1, Fpz, Fp2, F7, F3, Fz, F4, F8, FT7, FC3, FCz, FC4, FT8, T7, C3, Cz, C4, T8, TP7, CP3, CPz, CP4, TP8, P7, P3, Pz, P4, P8, PO5, POz, PO6, O1, Oz, O2, CB1 and CB2; Figure 1D) and two additional electrodes placed on the left and right mastoids (M1 and M2). An electrode on the tip of the nose served as online reference. To control for eye movements, the electrooculogram (EOG) was also monitored with two bipolar electrodes placed above and below the left eye (VEOG), and two bipolar electrodes placed on the outer canthi of the eyes (HEOG). The scalp electrodes were mounted on an elastic nylon cap (Quickcap, Neuroscan, Compumedics, Charlotte, NC, USA), in accordance with the extended 10–20 system. EEG signals were amplified using SynAmps RT amplifier (NeuroScan, Compumedics, Charlotte, NC, USA), digitized with a sampling rate of 1000 Hz, and online-low-pass filtered to 200 Hz using Neuroscan 4.4 software (NeuroScan, Compumedics, Charlotte, NC, USA). During the acquisition, all electrode impedances were kept below 10 kΩ.

The electrocardiogram and the skin conductance response (SCR) data were acquired using AcqKnowledge 4.2 software and Biopac MP150 acquisition system (Biopac Systems Inc., Goleta, CA, USA), with a sampling rate of 1000 Hz. Electrocardiogram data were collected using the Biopac ECG100C-MRI amplifier (Biopac Systems Inc., Goleta, CA, USA), with disposable Ag–AgCl electrodes aligned in a standard configuration (Right and Left sides of the body, under the rib and right sternum, just below the clavicle). SCR was obtained using the Biopac EDA100C-MRI amplifier (Biopac Systems Inc., Goleta, CA, USA) with the electrodes placed on the upper phalange of the middle and index fingers of the left hand.

### Data Analysis

Data from 25 scalp electrodes (F7, F3, Fz, F4, F8, FT7, FC3, FCz, FC4, FT8, T7, C3, Cz, C4, T8, TP7, CP3, CPz, CP4, TP8, P7, P3, Pz, P4, P8), and from HEOG and VEOG channels were analyzed using EEGLAB (Delorme and Makeig, 2004) and Fieldtrip toolboxes (Oostenveld et al., 2011) running on MATLAB. EEG responses were re-referenced to the average of all scalp electrodes, and filtered from 0.1 Hz to 200 Hz. Eye blinks, large saccades and other muscular artifacts were removed from the data by means of Independent Component Analysis (ICA), with the Second Order Blind Identification (SOBI) method (Delorme and Makeig, 2004; Delorme et al., 2007). Since we were interested in induced activity within the EEG gamma band, among others, we followed recommendations from Keren et al. (2010) when performing ICA artifact rejection, to avoid the contribution of micro-saccade muscular activity to gamma activity at the scalp level (Yuval-Greenberg and Deouell, 2011).

Four epochs of 60 s were defined for analysis of EEG data after disregarding the initial 30 and 60 s of the noise and the beat parts, respectively (Figure 1C), to allow the signal to stabilize. The resulting first epoch of each block was considered as “Baseline”, the two subsequent epochs, with binaural or acoustic beat stimulation, were called “Beat1” and “Beat2”, and the last epoch in the run was considered as post-treatment, and hence called “Post”. For the electrocardiogram and SCR data, epochs lasted 50 s, with the noise epochs (Baseline and Post) starting 5 s after and ending 5 s before the pink noise stimulation, and with the two beat epochs arranged as Beat1, which started 10 s after the onset of the beat, and Beat2 ending 10 s before the beat offset (Figure 1C).

For each of the EEG epochs, Baseline, Beat1, Beat2 and Post, data were analyzed in the spectral domain, separately for each block and electrode by means of the Fast-Fourier Transform (FFT; Slepian windowed). Separated EEG spectra were calculated for the two epochs of the beat, Beat1 and Beat2, ensuring the frequency resolution in the FFT was identical for the noise and beat epochs. Yet, results from these two beat epochs were averaged together into a single epoch (Beat). In each of the three resulting EEG spectra, Baseline, Beat and Post, for each block, power values were obtained from a window of 3.5 Hz centered at the frequencies of interest, corresponding to the frequencies of the beat stimulation in each block: 4.53 Hz (theta-beat), 8.97 Hz (alpha-beat), 17.93 Hz (beta-beat), 34.49 Hz (gamma-beat) or 57.3 Hz (upper gamma-beat). Power values within these frequency ranges were normalized by means of the following formula:

$$Freq_{dB}\;=\;10*\text{log}\left(\frac{Pi}{Pa}\right)$$

where Pi is the power value of the frequency of interest in the corresponding block, and Pa is the average power of the frequency of interest in the remaining blocks, for the same epoch (Baseline, Beat or Post) and condition (binaural-beat or acoustic-beat). For example, EEG power in the defined alpha window during the first noise epoch (Baseline) of the binaural alpha-beat block was divided by the average of power in alpha window in the first noise epoch of binaural theta-, beta-, gamma- and upper-gamma-beat blocks. Then, the resulting value was transformed into dB. By means of this transformation, we obtained a normalized power value for each epoch (Baseline, Beat, Post) and for each condition (binaural, acoustic) in each frequency of interest.

Scalp distribution of spectral power within each EEG frequency-window studied (theta, alpha, beta, gamma and upper-gamma) were obtained for illustrative purposes, and analyzed by taking averaged power values within two anterior (one left one right) and two posterior clusters of three electrodes each (F3, FC3, C3; F4, FC4, C4; CP3, P3, O1; CP4, P4, O2). Data from spectral EEG topographies were normalized by dividing the amplitude at each electrode by the sum of squared voltages at all electrodes analyzed per participant and per condition (McCarthy and Wood, 1985). With these analyses, we aimed to disentangle whether binaural beats at each frequency could induce changes in the scalp distribution within EEG bands.

As for the psychophysiological measures, heart rate was calculated from electrocardiogram data as the mean instantaneous heart rate averaged in 50-s time windows with no overlap, resulting in two values during noise epochs (Baseline and Post), and two values, during beat epochs (Beat1 and Beat2), averaged together (Beat). Similarly, we calculated the mean amplitude of the SCR, measured in microSiemens, in 50-s time windows with no overlap, resulting in two values during noise epochs (Baseline and Post), and two values, during beat epochs (Beat1 and Beat2), averaged together (Beat).

### Statistical Analysis

Statistical comparisons of normalized EEG spectral power were performed, separately for each of the frequency ranges studied, by means of a three-way repeated measures analysis of variance (ANOVA), with the three factors being Session (three levels: Baseline, Beat, Post), Treatment (two levels: binaural, acoustic) and Electrode (15 levels: 15 electrodes). Similarly, statistical comparisons of heart rate and SCR averages were performed by means of a two-way repeated measures ANOVA, with the two factors being Session (three levels: Baseline, Beat, Post) and Treatment (two levels: binaural, acoustic). We examined Session effects, with significant interactions between Session and Treatment factors.

Analyses of scalp distribution of EEG spectral power were performed, separately for each of the frequency ranges studied, by means of a repeated measures ANOVA with four factors: Session (three levels: Baseline, Beat, Post), Treatment (two levels: binaural, acoustic), Frontality (two levels: anterior or posterior clusters) and Laterality (two factors: left and right clusters).

For EEG spectral power, we aimed to compare the power within the frequency range of interest in the noise epochs with that in the epochs of beat stimulation, separately for acoustic- and binaural-beat conditions. For each frequency of interest, and for each type of beat (binaural or acoustic), increased EEG power in the defined frequency range during epochs of beat stimulation, in comparison of epochs of noise, would indicate an enhancement effect of the stimulation. An interaction with the Treatment factor would indicate if that effect is attributable to the binaural beat. The same rationale applied for changes in heart rate and the SCR.

For each of the comparisons, whenever the assumption of sphericity was violated, degrees of freedom were corrected using Greenhouse-Geisser estimates. The alpha level was set to 0.05. Effect size and observed power values were calculated for each statistical test. For all the statistical analyses, Bonferroni corrections for multiple comparisons were performed to adjust the *p*-values.

## Results

### EEG Frequency Analysis

**Figure 2:**
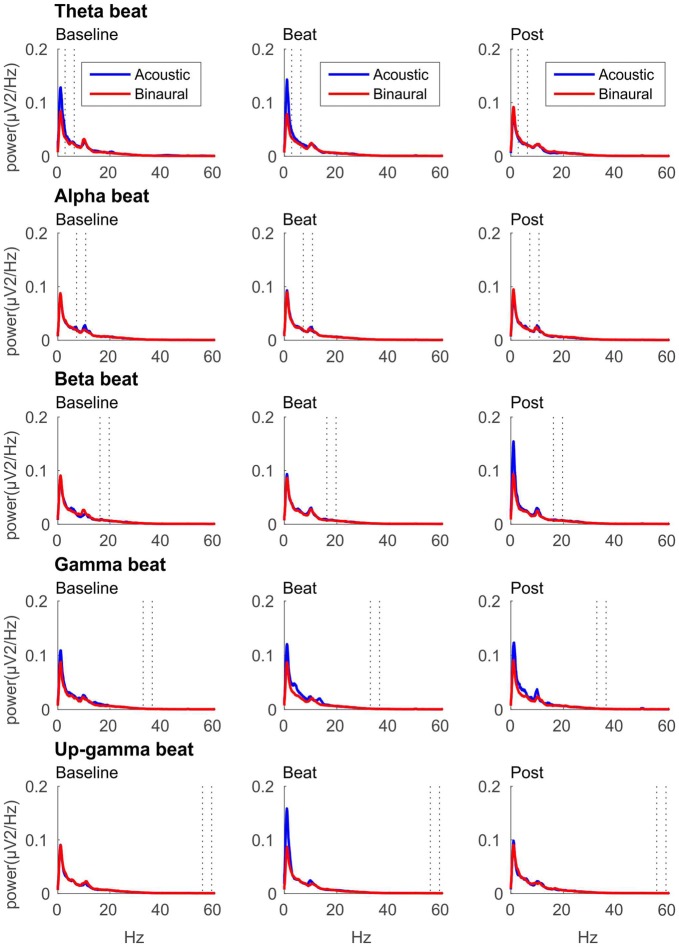
EEG spectra for each epoch of the block (three columns, from left to right: Baseline, Beat, Post) and each of the five beat frequencies of stimulation (five rows, from top to bottom: theta, alpha, beta, gamma and upper gamma), before normalization. Red lines correspond to EEG spectra obtained in binaural-beat blocks, and blue lines in acoustic-beat blocks. Dotted lines in each spectrum show the frequency window from which power values were obtained and normalized for statistical analyses.

If brainwave entrainment at the specific frequency of the binaural beat had occurred, we would observe a power enhancement at that specific frequency in the EEG spectrum when binaural beats were presented. However, such power enhancement was not observed for any of the tested beat frequencies, as well as no differences were observed between the EEG spectra obtained for binaural and acoustic beat conditions in the epochs of auditory stimulation. For illustrative purposes, in Figure 2 we include the EEG spectrum obtained for each epoch of the block (Baseline, Beat, Post) and each of the five beat frequencies used (theta, alpha, beta, gamma and upper gamma).

After normalization, as summarized in Table 1, no significant effects whatsoever were found in the EEG spectral power within the theta, alpha, beta or gamma frequency ranges analyzed. Specifically, ANOVA analyses yielded no effect for the Session factor, indicating no differences in the normalized spectral power values, within the EEG bands analyzed, between the noise and beat epochs of the block. No significant effects were found for the Treatment factor neither, indicating no overall differences between binaural-beat and acoustic-beat stimulation. Furthermore, no interaction between Session and Treatment factors were found. According to these results, no enhancement of EEG spectral power would be induced neither with binaural or with acoustic beats of the reported frequencies. With binaural beats in the upper gamma frequency, which cannot be perceived (Licklider et al., 1950), EEG spectral power in the upper gamma frequency range analyzed showed no significant increase in relation to acoustic beats.

**Table 1:** Results of analysis of variance (ANOVA) on normalized values of electroencephalographic (EEG) power within theta (2.78–6.28 Hz), alpha (7.22–10.72 Hz), beta (16.18–19.68 Hz), gamma (32.74–36.24 Hz) and upper-gamma (55.55–59.05 Hz) bands.

Beat frequency	*df*	*F*	*p*	$\eta_{\text{p}}^{2}$	Observed power^a^
**Theta**					
Session	2, 22	0.641	0.536	0.055	0.144
Treatment	1, 11	0.662	0.433	0.057	0.116
Session*Treatment	2, 22	1.195	0.180	0.017	0.076
Electrode	24, 264	0.641	0.903	0.055	0.544
**Alpha**					
Session	2, 22	2.358	0.115	0.154	0.434
Treatment	1, 13	1.010	0.333	0.072	0.154
Session*Treatment	2, 26	0.611	0.551	0.045	0.141
Electrode	24, 264	3.253	**>0.001**	0.200	1
**Beta**					
Session	2, 26	0.594	0.559	0.044	0.138
Treatment	1, 13	0.026	0.874	0.002	0.053
Session*Treatment	1.22, 15.96	0.554	0.582	0.041	0.132
Electrode	24, 264	0.641	0.904	0.047	0.550
**Gamma**					
Session	1.43, 18.6	0.019	0.981	0.001	0.053
Treatment	1, 13	0.081	0.781	0.006	0.058
Session*Treatment	2, 26	1.273	0.297	0.089	0.251
Electrode	3.08, 40.05	1.401	0.254	0.097	0.364
**Upper-gamma**					
Session	1.43, 18.6	1.185	0.312	0.084	0.236
Treatment	1, 13	0.811	0.384	0.059	0.133
Session*Treatment	2, 26	0.089	0.915	0.007	0.062
Electrode	24, 264	2.743	0.054	0.174	0.628

Session factor includes three levels refering to the different epochs of the block (Baseline, Beat and Post), whereas Treatment factor refers to binaural or acoustic beat condition. Session*Treatment refers to the interaction between them. Electrode factor includes 25 levels for the 25 electrodes analyzed. For each factor and their interaction, degrees of freedom (df), F and p values, size effects ($\eta_{\text{p}}^{2}$) and observed power are presented. P values below 0.05 are highlighted in bold. *P* values in the table are non-corrected for multiple comparisons. ^a^calculated for alpha = 0.05.

**Figure 3:**
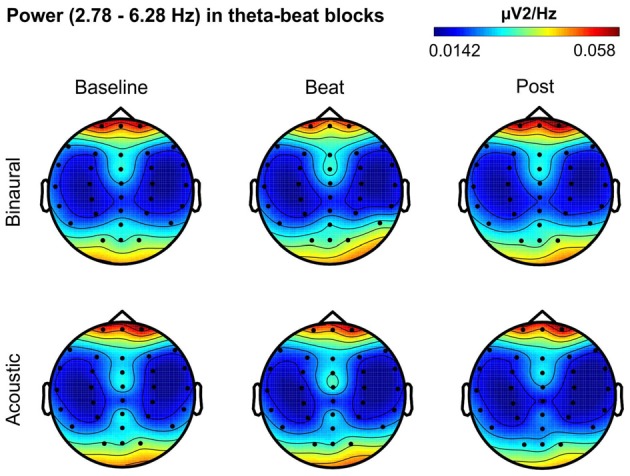
Scalp distribution of EEG spectral power during blocks with theta-beat stimulation. From left to right, scalp maps correspond to Baseline, Beat, Post epochs of the blocks, respectively. On top, scalp maps in blocks with binaural-beat stimulation. At the bottom, scalp maps in blocks with acoustic-beat stimulation. EEG spectral power corresponds to a frequency window of 3.5 Hz around the frequency of stimulation (4.53 Hz). Black dots correspond to electrode positions.

**Figure 4:**
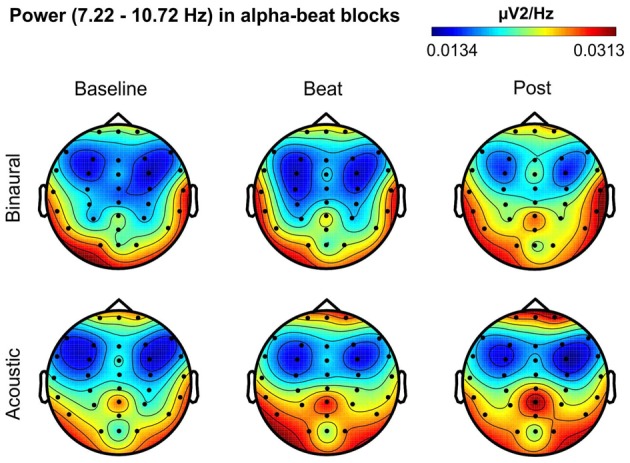
Scalp distribution of EEG spectral power during blocks with alpha-beat stimulation. From left to right, scalp maps correspond to Baseline, Beat, Post epochs of the blocks, respectively. On top, scalp maps in blocks with binaural-beat stimulation. At the bottom, scalp maps in blocks with acoustic-beat stimulation. EEG spectral power corresponds to a frequency window of 3.5 Hz around the frequency of stimulation (8.97 Hz). Black dots correspond to electrode positions.

**Figure 5:**
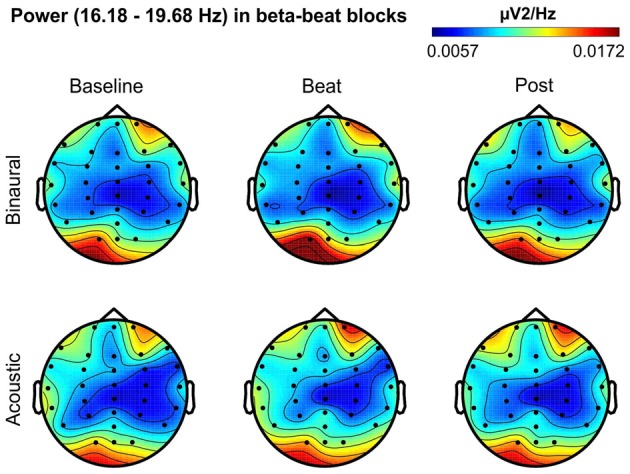
Scalp distribution of EEG spectral power during blocks with beta-beat stimulation. From left to right, scalp maps correspond to Baseline, Beat, Post epochs of the blocks, respectively. On top, scalp maps in blocks with binaural-beat stimulation. At the bottom, scalp maps in blocks with acoustic-beat stimulation. EEG spectral power corresponds to a frequency window of 3.5 Hz around the frequency of stimulation (17.93 Hz). Black dots correspond to electrode positions.

**Figure 6:**
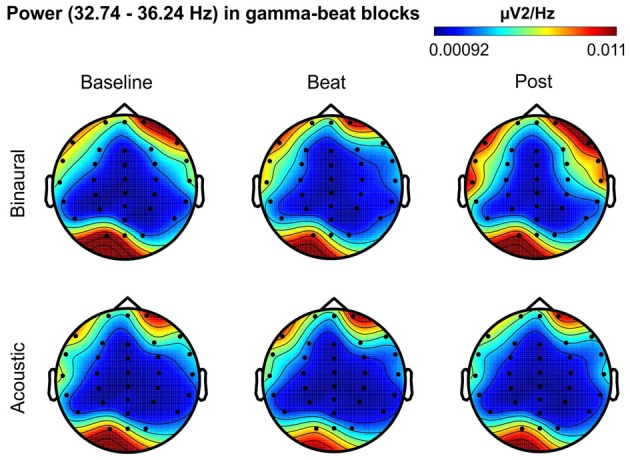
Scalp distribution of EEG spectral power during blocks with gamma-beat stimulation. From left to right, scalp maps correspond to Baseline, Beat, Post epochs of the blocks, respectively. On top, scalp maps in blocks with binaural-beat stimulation. At the bottom, scalp maps in blocks with acoustic-beat stimulation. EEG spectral power corresponds to a frequency window of 3.5 Hz around the frequency of stimulation (34.49 Hz). Black dots correspond to electrode positions.

**Figure 7:**
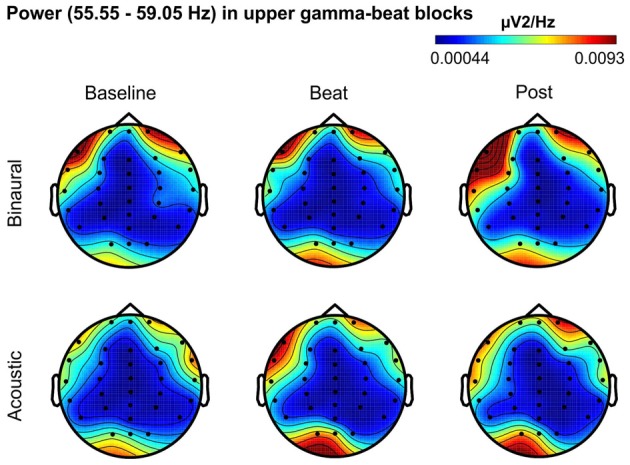
Scalp distribution of EEG spectral power during blocks with upper-gamma-beat stimulation. From left to right, scalp maps correspond to Baseline, Beat, Post epochs of the blocks, respectively. On top, scalp maps in blocks with binaural-beat stimulation. At the bottom, scalp maps in blocks with acoustic-beat stimulation. EEG spectral power corresponds to a frequency window of 3.5 Hz around the frequency of stimulation (57.3 Hz). Black dots correspond to electrode positions.

Besides changes in EEG spectra, we also examined differences in the spectral EEG topographies among conditions. In Figures 3–7, we show the scalp distribution of EEG spectral power within the theta, alpha, beta, gamma and upper gamma bands studied, respectively, for the Baseline, Beat and Post epochs of the blocks. For this analysis, we used two anterior and two posterior clusters of electrodes, aiming to explore interactions between Session or Treatment factors with different regions of the scalp (studied through Frontality and Laterality factors). If binaural beats would affect the distribution of EEG spectral power across the scalp, we would observe an interaction between Session and Treatment factors, and Frontality and/or Laterality factors. That is, changes in spectral power would be different across different Frontality or Laterality levels, thus revealing different topographical distributions of EEG spectral power. However, no significant interactions between Session or Treatment, and Frontality or Laterality factors, were found within any of the EEG bands tested. In topographies within alpha band, only two triple interactions between Session, Treatment and Frontality (*F*_(2,24)_ = 3.829, *p* = 0.036), and between Session, Frontality and Laterality (*F*_(2,24)_ = 4.055, *p* = 0.030) yielded *p* values below 0.05 which, however, did not survive the Bonferroni correction for the multiple comparisons. The same occurred for topographies within upper gamma band, where an interaction between Session and Laterality (*F*_(2,24)_ = 4.891, *p* = 0.017) vanished out after the Bonferroni correction.

### Psychophysiological Measures

As summarized in Table 2, analyses on the two psychophysiological measures studied yielded negative results for beta- and gamma-beat stimulation, as well as for the upper gamma condition. ANOVA showed no effects of Session or Treatment factors, as well as no interaction between them, neither for heart rate or for skin conductance measures. With regard to the SCR for theta- and alpha-beats, the ANOVA yielded *p* values below 0.05 for the Session factor (Table 2) which, nevertheless, vanished out after the Bonferroni correction for the multiple comparisons.

**Table 2:** Results of ANOVA on heart rate and skin conductance response (SCR; microSiemens) values in blocks of theta (4.53 Hz), alpha (8.97 Hz), beta (17.93 Hz), gamma (34.49 Hz) and upper-gamma (57.3 Hz) stimulation.

Heart rate	*df*	*F*	*p*	$\eta_{\text{p}}^{2}$	Observed power^a^
**Theta-beat**					
Session	2, 26	2.560	0.097	0.165	0.466
Treatment	1, 13	0.314	0.585	0.024	0.082
Session*Treatment	2, 26	0.883	0.425	0.064	0.185
**Alpha-beat**					
Session	2, 26	0.950	0.400	0.068	0.197
Treatment	1, 13	1.586	0.230	0.109	0.215
Session*Treatment	2, 26	0.049	0.952	0.004	0.057
**Beta-beat**					
Session	2, 26	0.640	0.535	0.047	0.145
Treatment	1, 13	0.993	0.337	0.071	0.152
Session*Treatment	2, 26	0.314	0.733	0.024	0.095
**Gamma-beat**					
Session	2, 26	0.156	0.854	0.012	0.072
Treatment	1, 13	0.603	0.451	0.044	0.111
Session*Treatment	2, 26	0.370	0.694	0.028	0.103
**Upper-gamma-beat**					
Session	2, 26	2.901	0.073	0.182	0.518
Treatment	1, 13	0.030	0.865	0.002	0.053
Session*Treatment	2, 26	1.514	0.239	0.104	0.293
**Skin conductance**					
**Theta-beat**					
Session	2, 26	3.934	**0.032**	0.232	0.655
Treatment	1, 13	2.164	0.165	0.143	0.276
Session*Treatment	2, 26	1.109	0.345	0.079	0.223
**Alpha-beat**					
Session	2, 26	4.845	**0.016**	0.271	0.751
Treatment	1, 13	0.001	0.980	0.000	0.050
Session*Treatment	1.39, 18.05	0.999	0.359	0.071	0.174
**Beta-beat**					
Session	2, 26	0.102	0.903	0.008	0.064
Treatment	1, 13	0.444	0.517	0.033	0.095
Session*Treatment	2, 26	0.294	0.748	0.022	0.092
**Gamma-beat**					
Session	2, 26	0.021	0.980	0.002	0.053
Treatment	1, 13	0.067	0.800	0.005	0.057
Session*Treatment	2, 26	1.411	0.262	0.098	0.275
**Upper-gamma-beat**					
Session	2, 26	1.225	0.310	0.086	0.243
Treatment	1, 13	0.655	0.433	0.048	0.117
Session*Treatment	2, 26	0.963	0.395	0.069	0.199

Session factor includes three levels refering to different epochs of the block (Baseline, Beat and Post), whereas Treatment factor refers to binaural or acoustic beat condition. Session*Treatment refers to the interaction between them. For each factor and their interaction, degrees of freedom (df), F and p values, size effects ($\eta_{\text{p}}^{2}$) and observed power are presented. P values below 0.05 are highlighted in bold. P values in the table are non-corrected for multiple comparisons. ^a^calculated for alpha = 0.05.

These results indicate that no changes occurred in these psychophysiological parameters between the noise and beat epochs the block, neither with binaural or with acoustic theta-, alpha-, beta-, gamma- or upper gamma-beats, as well as there were no overall differences between the effects of the two types of beats.

**Figure 8:**
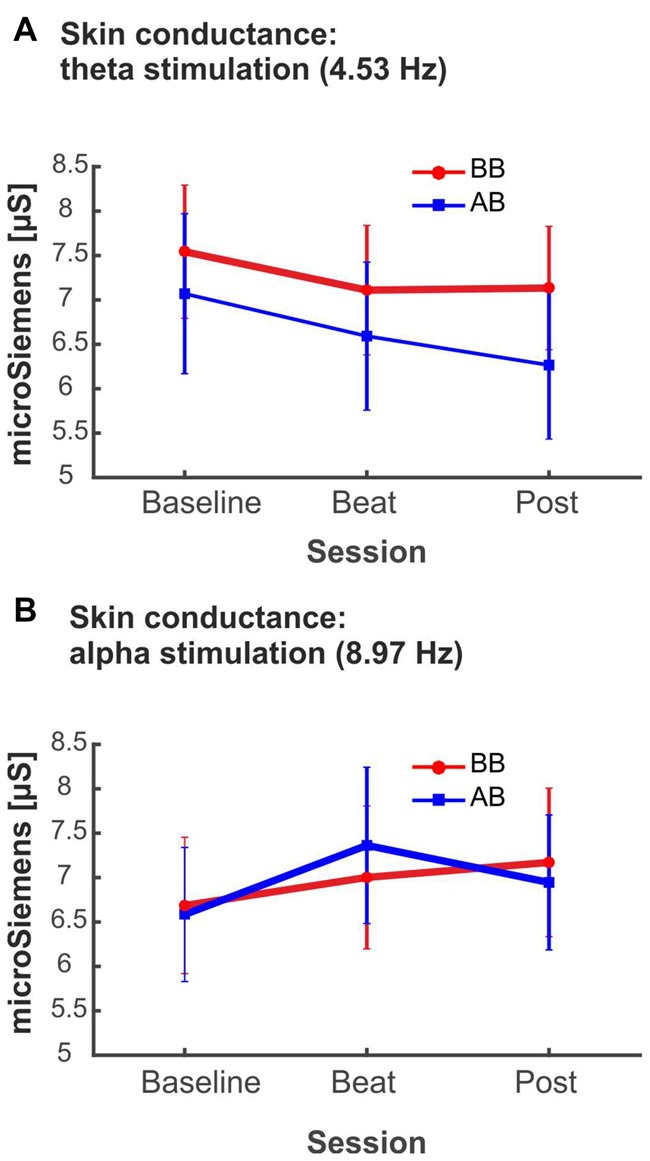
**(A)** SCR (microSiemens) during blocks with binaural beats (BB; red) or acoustic beats (AB; blue) of theta frequency (4.53 Hz). **(B)** SCR (microSiemens) during blocks with BB (red) or AB (blue) of alpha frequency (8.97 Hz). Error bars show the standard error of the mean.

Despite being not statistically significant, it is interesting to notice that, in the case of alpha-beat stimulation, skin conductance increased along the block, contrary to what would be expected if we consider increased SCR as associated to stress (see Figure 8A). In contrast, there was a decrease in skin conductance along the block with theta-beat stimulation, suggesting a relaxation effect with beats in this range, independently of the type of beat (see Figure 8B). Such trends along the session occur only with the two slowest beat frequencies studied here, theta and alpha, the typical beat frequencies reported in previous studies as related to clinically relevant psychophysiological dimensions (Padmanabhan et al., 2005; McConnell et al., 2014; Palaniappan et al., 2015).

## Discussion

### Summary of Results

The main goal of the present study was to disentangle the specific contribution of binaural-beat stimulation at different beat frequencies on specific EEG frequency bands. Particularly, we aimed to explore binaural beat effects on EEG power. Results yielded no effects of binaural beats in the theta, alpha, beta, gamma and upper gamma beat frequencies in the enhancement of EEG spectral power in their corresponding frequencies; similar negative results were also obtained with acoustic beats. Additionally, no effects from binaural or acoustic beats in the spectral EEG topographies were found either. As a secondary objective, we aimed to study whether binaural-beat stimulation would induce changes in two psychophysiological parameters, heart rate and skin conductance. Both binaural and acoustic beats produced no effect on these measures, although theta and alpha beats, independently on whether they were acoustic or binaural, yielded changes in skin conductance, yet vanishing out after multiple-comparison correction.

### Effects of Binaural Beats on Brain Rhythms

Previous attempts to study binaural beats have hypothesized that oscillatory brain activity recorded through EEG could be modulated by binaural beats of specific frequencies. Particularly, we focused on studies where binaural beats would induce changes in EEG power. The modulation of EEG rhythms would be genuinely generated by the binaural beat and would be distinct from the one produced with an acoustic beat, as binaural beats are generated within the central auditory system. Our results for all the beat frequencies tested do not support this hypothesis, and suggest no enhancement of EEG spectral power within classical EEG bands is induced with binaural beats. Using beats in the theta frequency range, with frequencies from 5 Hz to 8.5 Hz, similar negative results were found by Stevens et al. (2003), Wahbeh et al. (2007) and, more recently, Gao et al. (2014), who failed to elicit an increase in theta EEG band. In all these studies, binaural beat stimulation had longer duration than in the present study, up to 4 h, yet they yielded similar negative results on EEG theta band. For beats with frequencies within the EEG alpha band, our results go in accordance with those of Gao et al. (2014) and Vernon et al. (2014) in finding no increase in EEG alpha band with binaural-beat stimulation. Similarly, for binaural beta-beats, although findings of increased performance in cognitive-demanding tasks suggested promising results, we failed to observe a power enhancement on the EEG beta band analyzed, in accordance with findings from previous studies (Goodin et al., 2012; Gao et al., 2014; Vernon et al., 2014).

Regarding binaural gamma-beats, our results within gamma band contrast with findings from Lavallee et al. (2011), which suggested enhancement of EEG gamma band relative to baseline using binaural beats. However, binaural beat frequencies used to achieve such results were in the beta range (15 Hz). Furthermore, Becher et al. (2015) reported an EEG spectral power increase with acoustic beats of 40 Hz, contrasting with our negative results with gamma-beats, with similar frequencies. Our results also contrast with others assessing binaural beats effects on cognitive functions associated with gamma band, such as selective attention (Gruber et al., 1999; Sokolov et al., 2004). In this regard, Reedijk et al. (2015) reported improvements in selective attention with binaural-beat stimulation at gamma frequency, but the present findings suggest the mechanism by which such improvements were obtained do not involve EEG gamma band enhancement. Finally, binaural beats in the upper gamma range (e.g., 57.3 Hz), exceeding the frequency limit for the perception of binaural beats (Licklider et al., 1950), seem unable to induce changes in the corresponding EEG spectral power.

One limitation to take into consideration for the lack of positive findings in the present study is the sample size (14 participants), which may be too small to yield a statistically significant result in the event that a real effect of binaural beats may exist. As reported in the results’ tables, the observed power of our ANOVA tests is overall small. Therefore, caution should be taken when interpreting the present negative results, as the sample size may account for the null effects reported.

Previous studies on event-related potential modulation and auditory steady-state responses suggest that entrainment effects occur within seconds of the binaural beat stimulation (Karino et al., 2006; Kasprzak, 2011). Additionally, as mentioned, in previous studies testing the same hypothesis binaural beats were presented for a longer duration obtaining similar negative results (Wahbeh et al., 2007; Goodin et al., 2012; Gao et al., 2014). Therefore, we consider unlikely that the lack of increased EEG spectral power within specific bands in the present study is related with the short duration of 3 min used here for beat stimulation. In this regard, as suggested by some authors (Becher et al., 2015), another important factor to consider when trying to induce changes in EEG oscillatory activity at a particular frequency is the use of continuous tones or short repetitive bursts of stimulation. Studies recording evoked responses to binaural beats typically used short bursts of binaural beat stimulation. For example, Schwarz and Taylor (2005) elicited a 40 Hz binaural beat auditory steady-state response with binaural beats presented in short bursts of 1200 ms, with intervals of 1200 ms; Draganova et al. (2008), similarly, found a 40 Hz auditory steady-state response using pure tones of 1000 ms duration and 2000 ms stimulus onset asynchrony, and Pratt et al. (2010) elicited event-related potential components followed by oscillations corresponding to 3 and 6 Hz binaural beats, using bursts of 2000 ms, with inter-stimulus intervals between 950 ms and 1050 ms. On the other hand, some other studies investigating induced EEG oscillatory activity with binaural beats (Wahbeh et al., 2007; Goodin et al., 2012; Gao et al., 2014; Vernon et al., 2014) used, like we did, continuous stimulation, and expected enhancement of power along the whole session. Thus, in the context of previous studies, the use of continuous presentation instead of short burst for stimulation seems appropriate, and is probably not related with the lack of effects observed here for the EEG bands studied.

Apart from results in the EEG spectra, spectral EEG topographies within theta, alpha, beta, gamma and upper gamma frequencies did not seem to be affected by binaural beats in their corresponding frequencies. This suggests that binaural beats do not have any impact in scalp distribution of EEG spectral power neither.

### Psychophysiological Measures

Our results on heart rate and skin conductance do no support the proposal suggesting an effect of binaural beats on measures related with emotional arousal. In contrast with previous findings on heart rate variability (Palaniappan et al., 2015), parasympathetic activation (McConnell et al., 2014) or anxiety (Padmanabhan et al., 2005), we did not find a specific effect of binaural beat on heart rate and skin conductance for any of the beat frequencies tested here. On the other hand, our results go in accordance with the findings of Chaieb et al. (2015) regarding diminishing effects of binaural-beat stimulation on anxiety levels. Thus, our results suggest no effects of binaural or acoustic beats on autonomic responses. A limitation to consider when interpreting such lack of effects, as in the case of EEG analyses, is the small observed power of our statistical tests, given our small sample size. Another possible limitation is the short duration of stimulation used in the present study (3 min), which highly contrasts with the length of the stimulation used in previous studies (e.g., 20 and 30 min in Padmanabhan et al., 2005; McConnell et al., 2014 respectively). Whereas the stimulation length used in the present study may be appropriate to investigate effects on the EEG, it could be insufficient to induce changes in psychophysiological parameters, such as heart rate and skin conductance.

## Conclusion

This study has provided a thorough research on the potential effects of binaural-beat stimulation on enhancing EEG activity on specific frequency bands. Our aim was to verify the theoretical assumption on the effects of binaural beats in both EEG rhythms and psychophysiological responses. The literature on the field was inconclusive: we reviewed studies on the effects of binaural beats in EEG oscillatory activity, as well as on the effects of binaural beats in measures related with autonomic responses. We performed an experimental design using rigorous methodological controls, with baseline-treatment-washout sessions and treatment vs. “placebo” condition (a beat with the same frequency, but generated acoustically).

No effects of binaural-beat stimulation on EEG spectral power occurred with beat frequencies belonging to theta, alpha, beta, or gamma EEG ranges, as well as with those belonging to upper gamma band. On the other hand, our measures of heart rate and skin conductance did not support the effect of binaural beats on emotional arousal. Thus, our results altogether do not support binaural beats as a potential brainwave entrainment tool, nor they suggest any beneficial effect on clinically relevant dimensions.

## Author Contributions

FL-C and CE designed the study, designed and carried out data analysis and wrote the manuscript. FL-C supervised data acquisition.

## Conflict of Interest Statement

The authors declare that the research was conducted in the absence of any commercial or financial relationships that could be construed as a potential conflict of interest.
